# Neuroprotective effects of an Nrf2 agonist on high glucose-induced damage in HT22 cells

**DOI:** 10.1186/s40659-019-0258-z

**Published:** 2019-09-21

**Authors:** Jiangpei Zhao, Lerong Liu, Xia Li, Lingxiao Zhang, Jing Lv, Xueli Guo, Hui Chen, Tongfeng Zhao

**Affiliations:** 1 Department of Neurology, The Six Affiliated Hospital of Sun Yat-Sen University, Guangzhou, Guangdong 510655 People’s Republic of China; 2 Department of Endocrinology, The Six Affiliated Hospital of Sun Yat-Sen University, Guangzhou, Guangdong 510655 People’s Republic of China; 3 Department of Geriatric Medicine, Zhejiang Hospital, Hangzhou, Zhejiang 310013 People’s Republic of China; 4 Department of Vascular Surgery, The First Affiliated Hospital of Zheng-zhou University, Zhengzhou, Henan People’s Republic of China; 5 Department of Nephrology, Henan Provincial People’s Hospital, Zhengzhou, Henan 450000 People’s Republic of China

**Keywords:** Sulforaphane, High glucose, Hippocampus, NF-E2-related factor 2, Nuclear factor-κB

## Abstract

**Background:**

Oxidative stress is the hallmark of diabetic encephalopathy, which may be caused by hyperglycaemic toxicity. We aimed to discover pharmacologic targets to restore redox homeostasis. We identified the transcription factor Nrf2 as such a target.

**Methods:**

HT22 cells were cultured in 25 or 50 mM d-glucose with various concentrations of sulforaphane (SFN) (from 1.25 to 5.0 μM). Cell viability was tested with the Cell Counting Kit-8 assay. Reactive oxygen species (ROS) production was detected with an inverted fluorescence microscope using the dichlorodihydrofluorescein-diacetate fluorescent probe. The expression of NF-E2-related factor 2 (Nrf2), haem oxygenase-1 (HO-1) and nuclear factor-κB (NF-κB) at the mRNA and protein levels was detected by reverse transcription quantitative polymerase chain reaction and western blotting.

**Result:**

We found that a high glucose concentration (50 mM) increased the generation of ROS, downregulated the expression of Nrf2/HO-1 and upregulated the expression of NF-κB. Moreover, HT22 cell viability significantly decreased after culture in high-glucose medium for 24, 48 and 72 h, whereas the activation of the Nrf2/HO-1 pathway using a pharmacological Nrf2 activator abrogated this high-glucose-induced toxicity.

**Conclusion:**

This study suggests that the activation of the Nrf2–ARE signalling pathway might be a therapeutic target for the treatment of diabetic encephalopathy.

## Background

Diabetes is a metabolic disorder characterized by hyperglycaemia and is associated with complications that affect almost every part of the body. Recent clinical evidence has shown that people with diabetes have a higher chance than healthy individuals of developing Alzheimer’s disease (AD) [1]. Spatial learning and hippocampal long-term potentiation are impaired in streptozotocin (STZ)-diabetic rats, and cognitive impairment is associated with increased neuronal loss in the hippocampus of these animals [2]. Senile plaques and neurofibrillary tangles are hallmarks of AD. It has been found that rodent diabetes models exhibit an increase in tau phosphorylation and amyloid-β (Aβ) accumulation, which might increase the formation of senile plaques and neurofibrillary tangles [3].

Although the aetiology of diabetes-associated cognitive decline is likely multifactorial, high-glucose-induced neuronal damage appears to be involved in the pathogenesis of the disease [4]. Elevated blood glucose has been reported to increase the accumulation of advanced glycosylation end products (AGEs) in the tissues of diabetic subjects, which has been demonstrated to promote Aβ aggregation and stabilize neurofibrillary tangles [5]. It has been reported that high-glucose conditions induce neuronal damage directly, as hippocampal neuronal cells cultured under high-glucose conditions exhibit irregularly shaped nuclei, edematous endoplasmic reticulum, enlarged mitochondria and an increased apoptosis rate [6]. Furthermore, high glucose levels are associated with an increase in oxidative stress and the inflammatory response, which play a significant role in the development of diabetic encephalopathy [7].

Nuclear factor erythroid 2-related factor (Nrf2), a transcription factor induced in response to oxidative stress, triggers the production of various antioxidant enzymes and phase II detoxification enzymes, including haem oxygenase-1 (HO-1), quinone oxidoreductases (NQO1) and superoxide dismutase (SOD) [8]. Under normal conditions, Nrf2 is retained in the cytoplasm by binding to kelch-like ECH-associated protein 1 (Keap1). Stimuli such as oxidative stress lead to the disruption of the Nrf2/Keap1 complex and translocate Nrf2 to the nucleus, where it binds to the antioxidant response element (ARE) and induces the production of a battery of endogenous antioxidant enzymes [9]. Previous studies have shown that Nrf2 plays a significant role in regulating neurogenesis, proliferation and the differentiation of neural progenitor cells [10]. Nrf2 protects neurons against Aβ toxicity and improves cognitive functions in transgenic AD mice [11]. The activation of Nrf2 signalling might be a promising strategy to prevent or delay the development of diabetic-associated cognitive decline.

Sulforaphane (SFN) is a type of isothiocyanate found in cruciferous vegetables. SFN has been demonstrated to protect against acute brain injuries and neurodegenerative diseases by promoting the nuclear translocation of Nrf2 and thus triggering intracellular defence responses to oxidative stress [12]. The aim of the study was to determine whether the disruption of Nrf2 signalling occurs in HT22 cells under high-glucose conditions. We hypothesized that activation of the Nrf2/HO-1 pathway using a pharmacological Nrf2 activator can attenuate oxidative stress and inflammation in HT22 cells under high-glucose conditions.

## Materials and methods

### Cell culture

The HT22 mouse hippocampal cell line was obtained from Jennio Biotech Co., Ltd. (China). The cells were cultured in Dulbecco’s modified Eagle’s medium (Gibco; Thermo Fisher Scientific, Inc., Waltham, MA, USA) supplemented with 10% foetal bovine serum (Biological Industry, Cromwell, CT, USA) and were divided into three groups: the normal control group (treated with 25 mM glucose), the high-glucose group (treated with 50 mM glucose) and the mannitol group (treated with 25 mM glucose + 25 mM mannitol). SFN (New England Biolabs, Ipswich, MA, UK) was added to the high-glucose group at various concentrations (1.25–5.0 μM) followed by incubation at 37 °C and 5% CO_2_ in a humidified atmosphere.

### Chemical reagents

Sulforaphane 140 (New England Biolabs, Ipswich, MA, USA) was dissolved in phosphate-141 buffered saline (PBS). Cells cultured in high-glucose medium were treated with different concentrations of SFN (1.25 μM, 2.5 μM, 5 μM) for 0 or 72 h.

### RNA extraction and reverse transcription quantitative polymerase chain reaction (RT-qPCR)

Total RNA was extracted from cells using TRIzol reagent (Takara, Otsu, Japan). cDNA was synthesized from the total RNA using Prime-Script™ RT regent Kits with gDNA eraser (Takara, Otsu, Japan) according to the manufacturer’s instructions. mRNA expression levels were measured using RT-qPCR on a Biosystems 7500 system (Applied Biosystems, Inc., Carlsbad, Cal, USA). The reaction mixture (10 μL) contained SYBR Select Master Mix (5 μL), cDNA (1 μL), and forward and reverse primers (0.5 μL). A two-temperature cycle of 95 °C for 10 s and 60 °C for 30 s was run and repeated for 40 cycles. The relative quantify of the sample transcripts was calculated using the ΔΔCq method with GAPDH as a reference. All samples are expressed as the mean. The primer sequences used are listed in Table 1.

**Table 1:** Primer sequences for reverse transcription quantitative polymerase chain reaction

Gene	Forward primers	Reverse primers
*Nrf2*	GAAATGATGTCCAAGGAGCAA	AAGACTTCAAGATACAAGGTGCTG
*NF*-*κB*	ACCCTGAAATCAAAGACAAAGAG	GAAATCCGTAGTTCGAGTAGCC
*HO*-*1*	TGACAGAAGAGGCTAAGACCG	GTGAGGACCCACTGGAGGA
*GAPDH*	ATTCAACGGCACAGTCAAGG	CACCAGTGGATGCAGGGAT

### Gel electrophoresis and western blotting

Cell lysates were prepared using radioimmunoprecipitation assay (RIPA) lysis buffer (CWBio, Beijing, China) in the presence of protease inhibitor cocktail (Thermo Fisher Scientific, Waltham, MA, USA). The protein concentrations of the cell lysates were quantified using a Pierce BCA Protein Assay Kit (Thermo Fisher Scientific, Waltham, MA, USA). Equal amounts of total protein were loaded in each well of a 12% sodium dodecyl sulfate polyacrylamide gel and subjected to electrophoresis. The proteins were then transferred onto polyvinylidene fluoride membranes (EMD Millipore, Billerica, MA, USA). The membranes were blocked with 5% non-fat milk and incubated with primary antibodies against GAPDH (10494-1-AP, ProteinTech), Nrf2 (ab62352, Abcam), HO-1 (ab13243, Abcam) and NF-κB (ab16502, Abcam) at 4 °C overnight, followed by incubation with horseradish peroxidase-conjugated secondary antibodies; detection was performed using ECL Plus western blotting detection reagents.

### Measurement of reactive oxygen species (ROS) generation

Cells were cultured in 6-well plates for 0 or 72 h, washed with phosphate-buffered saline (HyClone; GE Healthcare, Logan, UT, USA) and incubated with 10 μM dichlorodihydrofluorescein-diacetate (DCFH-DA) fluorescent probe (Beyotime, Beijing, China) in serum-free medium for 30 min at 37 °C. Subsequently, the cells were examined using an inverted fluorescence microscope (Olympus Corporation, Tokyo, Japan).

### CCK8 assay

HT22 cells in the logarithmic growth phase were plated in 96-well plates at a density of 4 × 10^4^ cells per well. Cell viability was estimated using the CCK-8 assay following the manufacturer’s instructions (Dojindo Molecular Technologies, Inc., Kumamoto, Japan). CCK-8 was added to each well 0, 24, 48 and 72 h after culturing, and then the cells were incubated for 3 h at 37 °C prior to measurement. The absorbance at 450 nm was detected using a microplate reader (Multiskan FC; Thermo Fisher Scientific, Inc.).

### Immunofluorescence

Cells were fixed with 4% paraformaldehyde, and 0.1% Triton-X (Sigma) was used for cell permeabilization. Then, the cells were blocked with 3% bull serum albumin (Beyotime, Beijing, China) for 1 h. After washing with PBS 3 times, a primary anti-Nrf2 rabbit antibody (ab62352, Abcam) was incubated with cells at 4 °C overnight, and then a secondary antibody conjugated to Cy3 (Multi Science, Hangzhou, China) was incubated with the cells at room temperature for 1 h. The fluorescence intensity was observed using laser scanning confocal microscopy (Leica TCS SP8, Leica Microsystems Inc., Buffalo Grove, USA).

### Measurement of IL-1β and IL-6

Cells were cultured in 6-well plates for 0 or 72 h, then washed with phosphate-buffered saline (HyClone; GE Healthcare, Logan, UT, USA). The concentrations of IL-1β and IL-6 were measured using Mouse IL-1β ELISA Kits (MULTI SCIENCES, Hangzhou, China, Cat. 70-EK201BHS/3-48) or Mouse IL-6 ELISA Kits (MULTI SCIENCES, Hangzhou, China, Cat. 70-EK206HS/3-48) according to the manufacturer’s protocol. Briefly, cell culture supernatants were collected after centrifuged at 300 g for 30 min and added 100 μL to per well in the testing plate coated with primary antibodies. After incubating in room temperature for 2 h, incubated in room temperature for 2 h, streptavidin conjugated with HRP was added to the wells, continuing to incubate in room temperature for 45 min. After that, TMB was added to the wells for color reactions after washing the wells with PBS (HyClone; GE Healthcare, Logan, UT, USA) for 3 times. The fluorescence intensity at 450 nm was measured using a microplate reader (Multiskan FC; Thermo Fisher Scientific, Inc.). The concentrations of IL-1β and IL-6 were calculated according to the standard curve.

### Statistical analysis

All experiments were replicated independently three times in triplicate. The values are expressed as the mean ± standard deviation. One-way analysis of variance combined with the least significant difference post hoc test was used to compare the mean values among the control and treatment groups by SPSS17.0 software (SPSS Inc., Chicago, IL, USA). Differences between the groups were considered to be statistically significant at P < 0.05.

## Results

### 1. High glucose levels inhibited the Nrf2/HO-1 pathway and activated the NF-κB pathway in HT22 cells

**Fig. 1:**
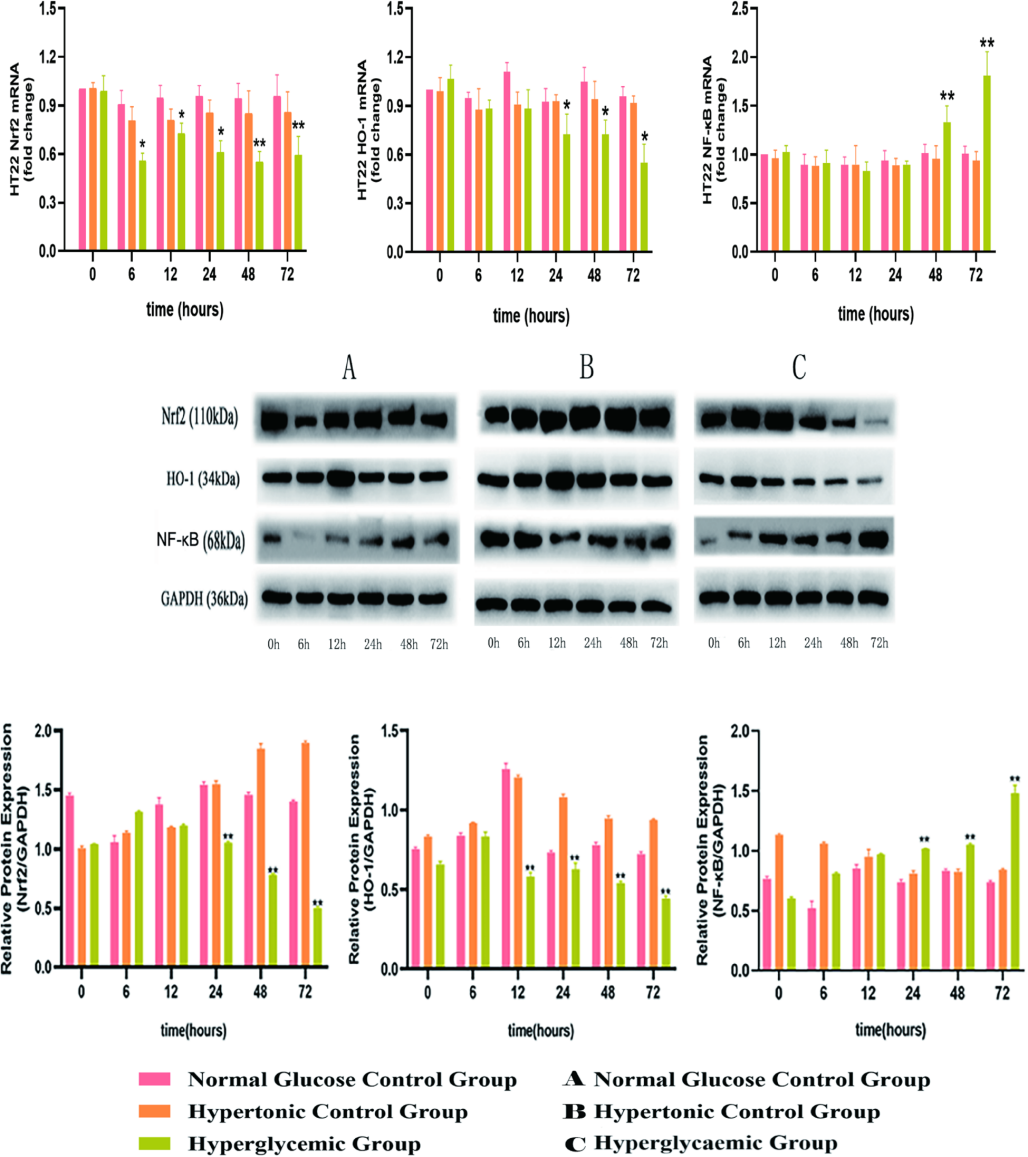
Effect of high glucose on Nrf2, HO-1 and NF-κB expression in HT22 cells. HT22 cells were cultured in normal glucose medium (25 mM d-glucose, A), hypertonic medium (25 mM glucose + 25 mM mannitol, B) or high-glucose medium (50 mM d-glucose, C) for 0, 6, 12, 24, 48 or 72 h. The mRNA and protein levels in the HT22 cells were assessed using reverse transcription quantitative polymerase chain reaction and western blotting. *P < 0.05, **P < 0.01 vs. the normal glucose control group; ^#^P < 0.05, ^##^P < 0.01 vs. the high-glucose control group. *Nrf2-1* NF-E2-related factor 2, *HO-1* haem oxygenase-1, *NF-κB* nuclear factor-κB

To investigate whether high-glucose medium affects oxidative stress and inflammatory status, HT22 cells were incubated with different concentrations of glucose for 0, 6, 12, 24, 48 and 72 h (Fig. 1).

High glucose treatment decreased the expression of Nrf2 mRNA at 6, 12, 24, 48 and 72 h. In addition, high glucose treatment also decreased the protein level of Nrf2. We further assessed the effect of high glucose on the Nrf2 downstream gene HO-1. The results showed that high glucose treatment significantly decreased the mRNA (24, 48, and 72 h) and protein expression (12, 24, 48, and 72 h) of HO-1 in a time-dependent manner. These observations confirmed that the Nrf2/HO-1 pathway was suppressed under high-glucose conditions. A recent study found that the Nrf2/HO-1 pathway interferes with the inflammation cascade and that the inhibition of this pathway can trigger inflammatory responses. NF-κB is a transcription factor that modulates the expression of inflammatory cytokines. We detected the expression levels of NF-κB to determine whether this pathway is activated when HT22 cells are cultured in high-glucose conditions. The results showed that high-glucose conditions increased the expression of NF-κB, which was most obvious at 48 and 72 h. There were no significant differences in Nrf2, HO-1 and NF-κB mRNA and protein levels between the control group and mannitol group.

### 2. High glucose levels increased the production of ROS in HT22 cells

**Fig. 2:**
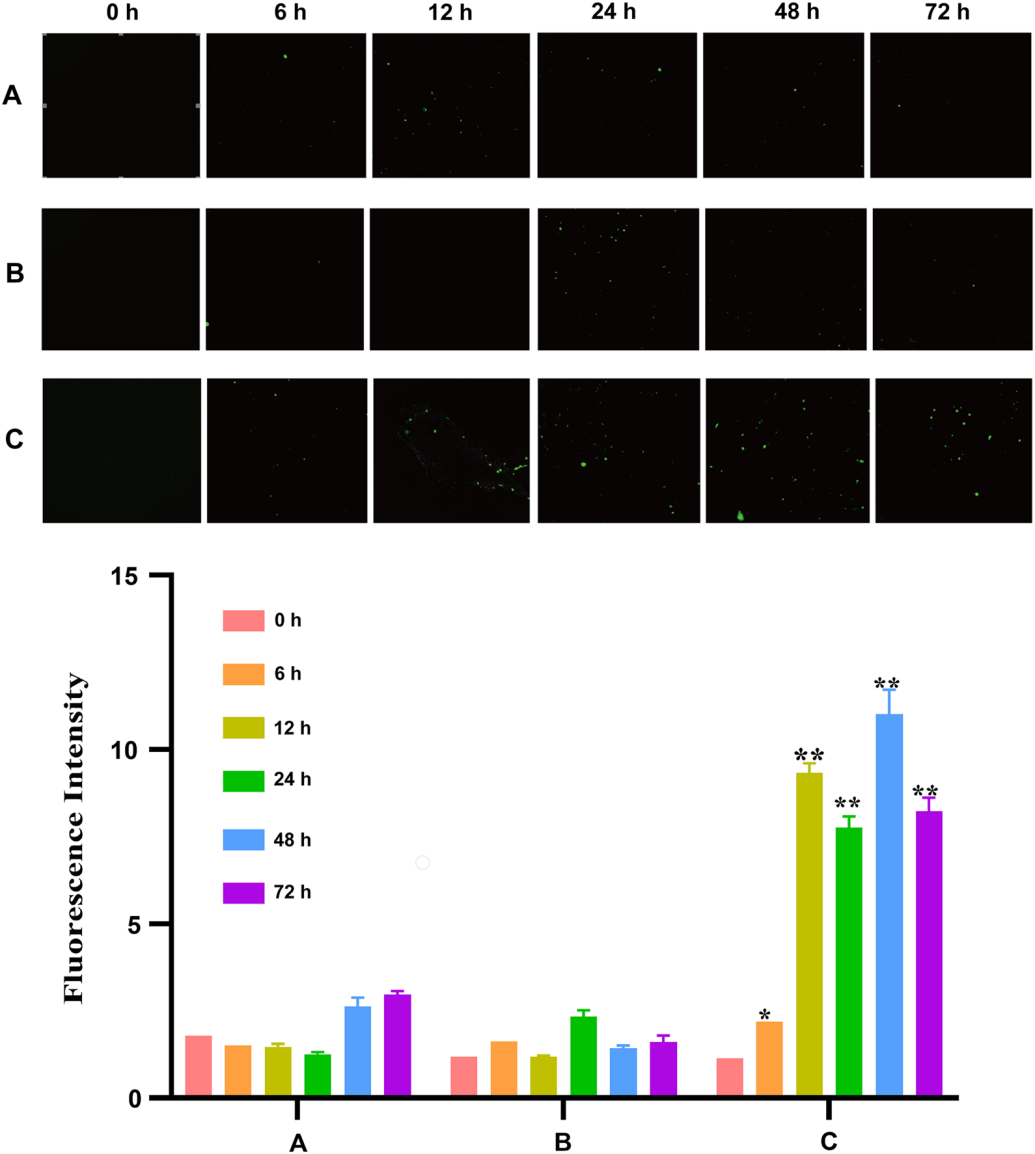
ROS production in cells cultured in different types of medium. HT22 cells were cultured in normal glucose medium (25 mM d-glucose, A), hypertonic medium (25 mM glucose + 25 mM mannitol, B) or high-glucose medium (50 mM d-glucose, C) for 0, 6, 12, 24, 48 or 72 h. ROS production was measured using the DCFH-DA fluorescent probe under a fluorescence microscope at a magnification of ×400 and semi-quantified using ImageJ. The fluorescence intensity was correlated with the ROS level. The fluorescence intensity was significantly increased over time in HT22 cells in the high-glucose medium group (C) compared with the hypertonic medium group (B) and normal glucose medium group (A). *ROS* reactive oxygen species, *DCFH-DA* dichlorodihydrofluorescein-diacetate

The intracellular ROS level was evaluated by detecting DCFH-DA fluorescence. The results showed that HT22 cells subjected to high glucose exhibited a significant increase in 2′,7′-dichlorofluorescein (DCF) fluorescence, and there was no significant difference in ROS production between the control group and mannitol group (Fig. 2).

### 3. SFN protected HT22 cells against high glucose-induced injury

**Fig. 3:**
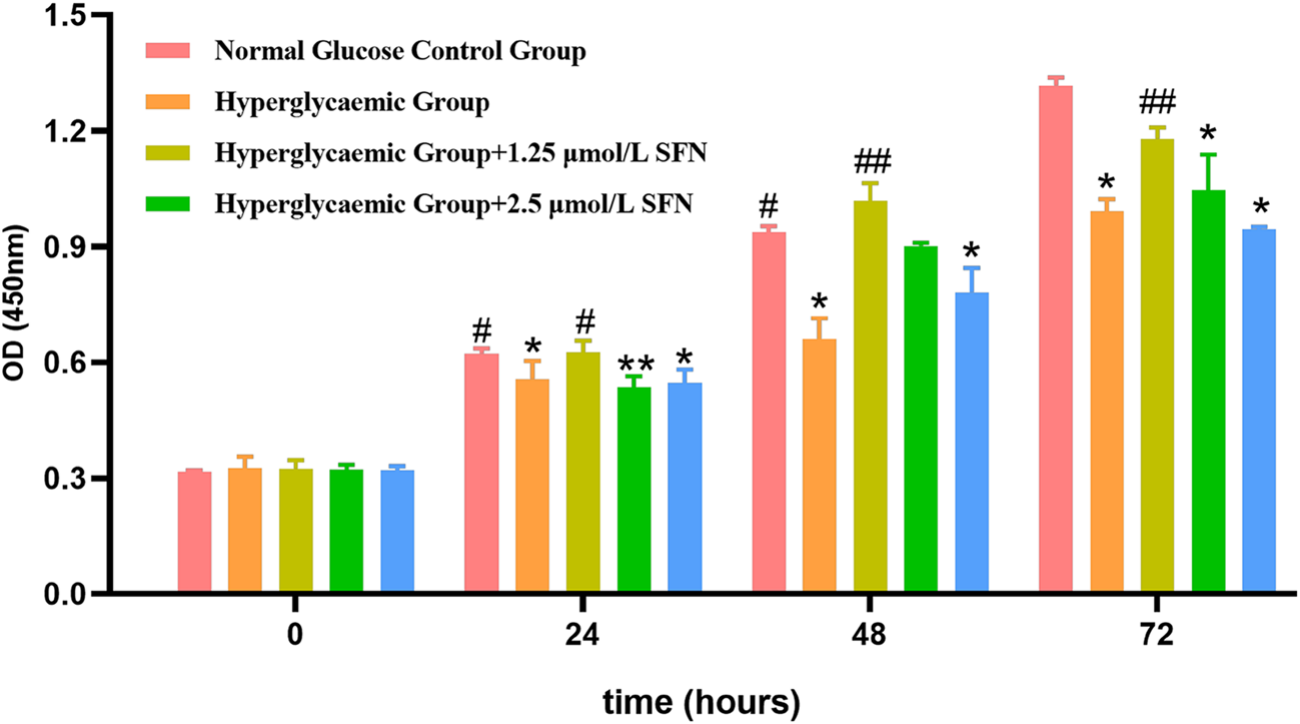
Effect of SFN on cell viability. HT22 cells were cultured in normal glucose medium or high-glucose medium under various concentrations of SFN for 0, 24, 48 or 72 h. Cell viability was measured using the CCK-8 assay as described. The values are expressed as the mean ± standard deviation (n = 3 for each group). *P < 0.05, **P < 0.01 vs. the normal glucose control group; ^#^P < 0.05, ^##^P < 0.01 vs. the high-glucose control group. *SFN* sulforaphane, *OD* optical density

Cell viability was determined by the CCK8 assay. We found that the cell viability of the HT22 cells was significantly decreased in the high-glucose group at 24, 48 and 72 h compared with that of the control group. To evaluate the cytoprotective effect of SFN against high-glucose conditions, HT22 cells were treated with 50 mM glucose and different concentrations of SFN (from 1.25 to 5.0 µM) for the indicated time periods. Treatment with 1.25 and 2.5 µM SFN ameliorated glucose toxicity and significantly increased cell viability in a time-dependent manner. Although 5.0 µM SFN increased the cell viability of the HT22 cells at 48 h, there was no significant difference at 24 or 72 h, and cell viability even decreased slightly at 72 h. These observations clearly demonstrate that SFN protects HT22 cells against high glucose-induced damage (Fig. 3).

### 4. SFN facilitated the intra-nuclear translocation of Nrf2

**Fig. 4:**
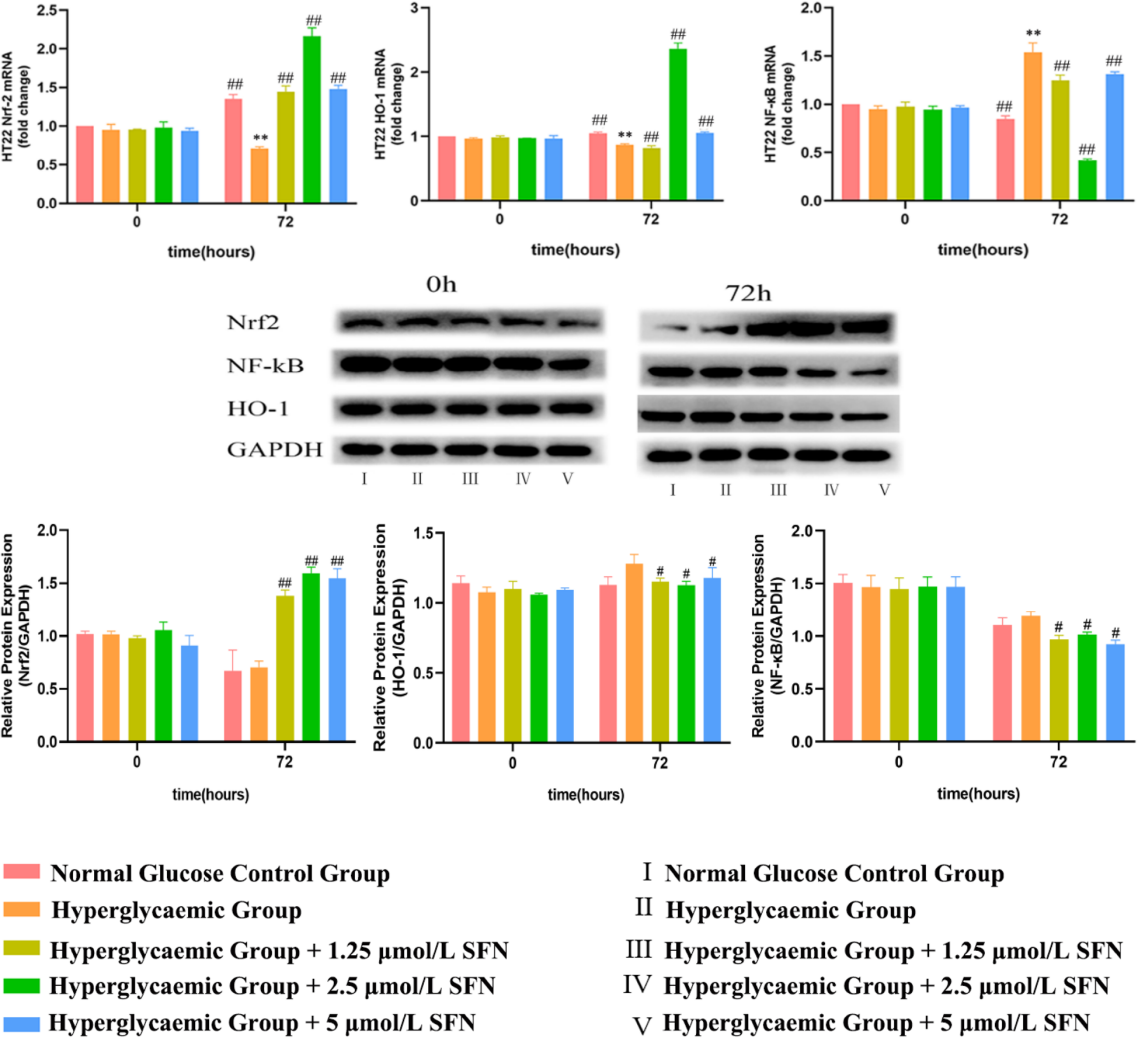
Effect of SFN on Nrf2, HO-1 and NF-κB expression in HT22 cells. HT22 cells were cultured in normal glucose medium or high-glucose medium with various concentrations of SFN for 0 or 72 h. The mRNA and protein levels in the HT22 cells were assessed using reverse transcription quantitative polymerase chain reaction and western blotting. The data are expressed as the mean ± standard deviation (n = 3 for each group). *P < 0.05, **P < 0.01 vs. the normal glucose control group; ^#^P < 0.05, ^##^P < 0.01 vs. the high-glucose control group. *SFN* sulforaphane, *Nrf2-1* NF-E2-related factor 2, *HO-1* haem oxygenase-1, *NF-κB* nuclear factor-κB

The fluorescence intensity of Nrf2 in the nucleus was reduced after high-glucose medium treatment in HT22 cells for 72 h, and treatment with SFN (from 1.25 to 5.0 µM) induced a significant increase in Nrf2 fluorescence in the nucleus. The results above suggest that SFN can increase Nrf2 translocation to the nucleus (Fig. 4).

### 5. SFN activated the Nrf2/HO-1 pathway and inhibited the NF-κB pathway in HT22 cells

**Fig. 5:**
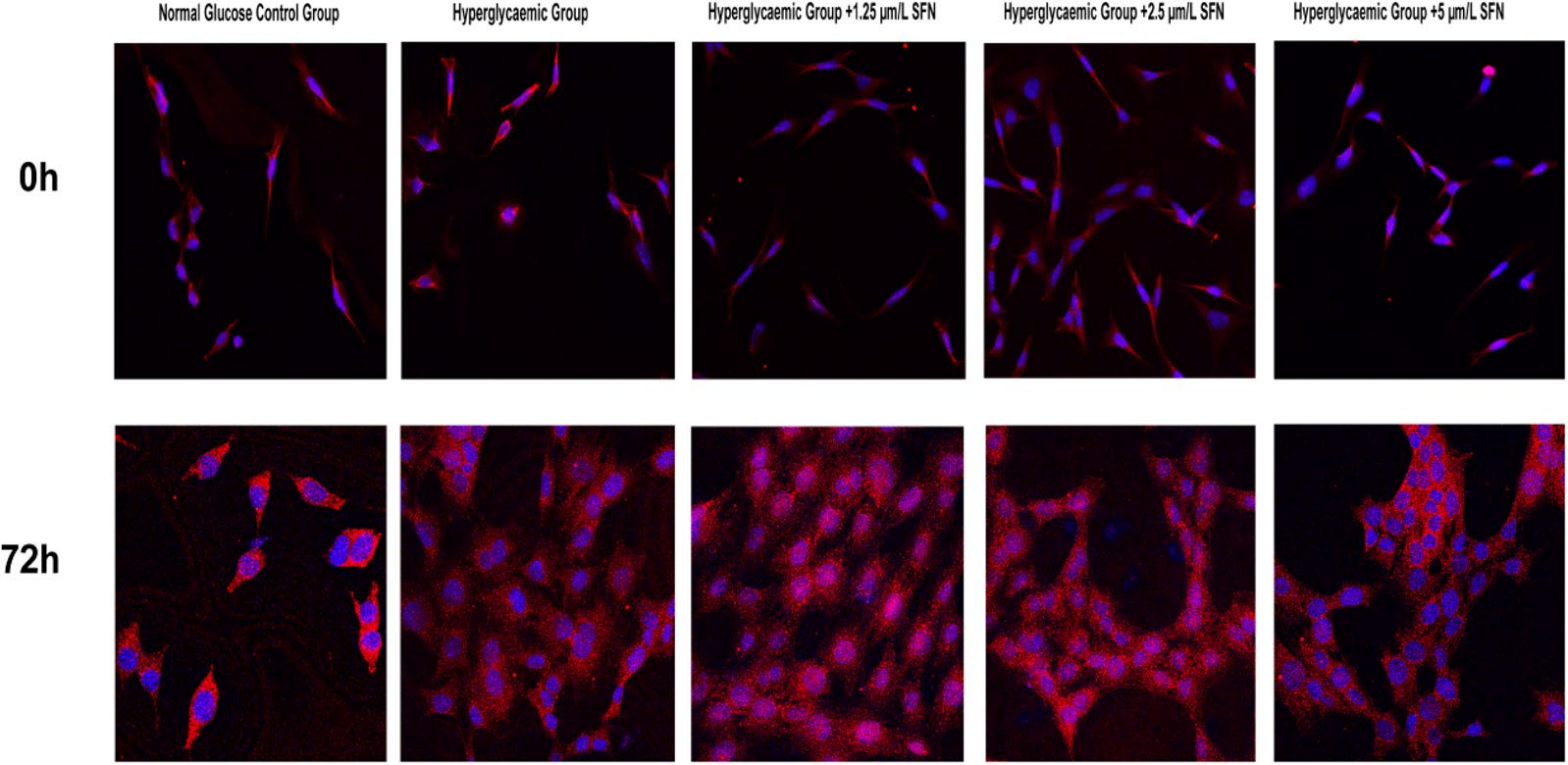
Nrf2 immunofluorescence. HT22 cells were cultured in normal glucose medium (25 mM d-glucose), high-glucose medium (50 mM d-glucose), high glucose-medium + 1.25 µm/L SFN, high-glucose medium + 2.5 µm/L SFN or high-glucose medium + 5 µm/L SFN for 0 or 72 h. Nrf2 fluorescence (Cy3) was measured by using a laser scanning confocal microscope at a magnification of ×200, and the excitation/emission wavelength was 488/532 nm. The fluorescence intensity in the nucleus was decreased in high-glucose medium but significantly increased in high-glucose medium with different concentrations of SFN after culturing for 72 h

To investigate the effect of SFN on oxidative stress and inflammation, we detected the expression of Nrf2, HO-1 and NF-κB using western blotting and RT-qPCR. Since the changes in cell viability and the expression of Nrf2, HO-1 and NF-κB were most notable after treatment with high glucose for 72 h, incubation with high glucose for 72 h was selected as the optimal condition for subsequent experiments. We found that SFN (from 1.25 to 5.0 µM) increased the expression of Nrf2 and HO-1 and decreased the mRNA and protein expression of NF-κB at 72 h in HT22 cells incubated with high glucose. The changes in Nrf2, HO-1 and NF-κB mRNA levels were most obvious after treatment with 2.5 µM SFN. The results above suggest that the activation of the Nrf2/HO-1 pathway using a pharmacological Nrf2 activator may suppress inflammation (Fig. 5).

### 6. SFN decreased the production of IL-1β and IL-6 in HT22 cells

**Fig. 6:**
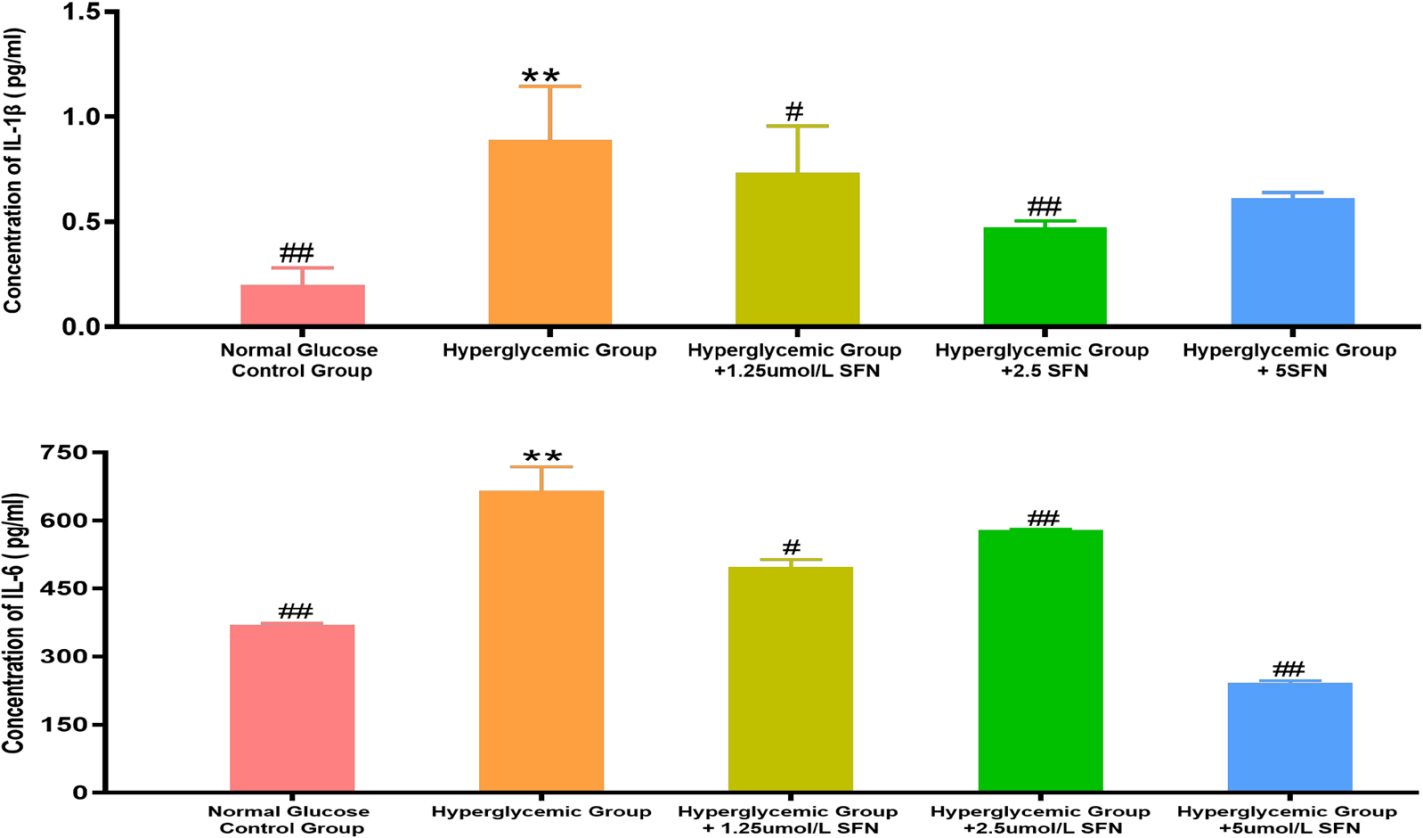
IL-1β and IL-6 concentrations in different cell culture supernatants. HT22 cells were cultured in normal glucose medium (25 mM d-glucose), high glucose medium (50 mM d-glucose), high glucose medium + 1.25 μm/L SFN, high glucose medium + 2.5 μm/L SFN or high glucose medium + 5 μm/L  SFN for 72 h. IL-1β and IL-6 levels were measured using the Mouse IL-1β ELISA Kit and Mouse IL-6 ELISA Kit under a fluorescence micro-reader at a wavelength of 450 nm. The fluorescence intensity was correlated with IL-1β and IL-6 concentrations and the concentrations of IL-1β and IL-6 were calculated according the standard curve. *P < 0.05, **P < 0.01 vs. Normal Glucose Control Group; ^#^P < 0.05, ^##^P < 0.01 vs. High Glucose Control Group

High glucose medium significantly increased the concentrations of IL-1β and IL-6 in cell culture supernatants of HT22 cells at 72 h, and treatment with SFN (from 1.25 to 5.0 µM) decreased the concentrations of IL-1β and IL-6 in the supernatants. The results above suggest that the activation of the Nrf2/HO-1 pathway using a pharmacological Nrf2 activator may inhibit the production of inflammatory cytokines induced by NF-κB (Fig. 6).

### 7. SFN decreased the production of ROS in HT22 cells

**Fig. 7:**
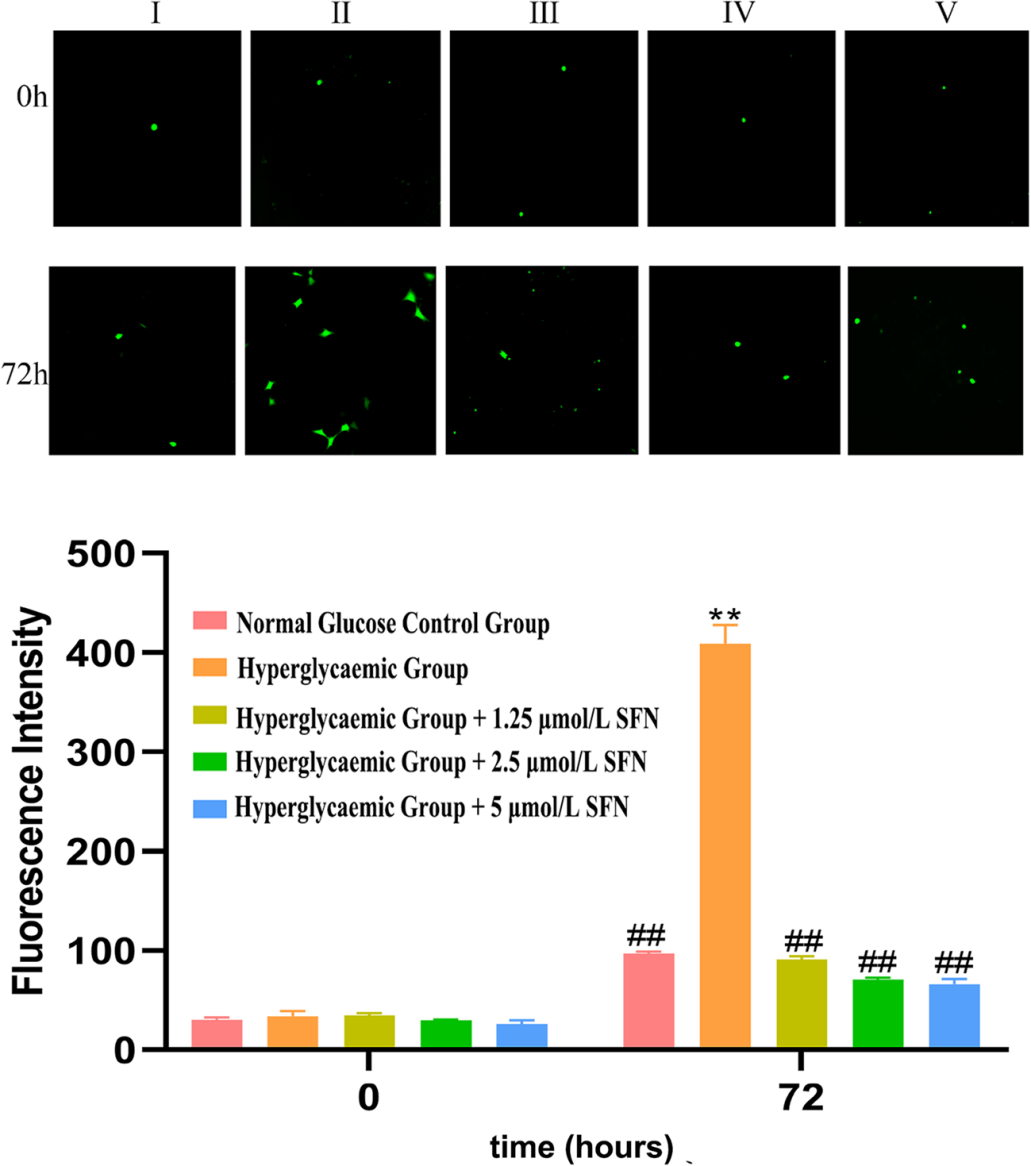
ROS production in cells cultured in different types of medium. HT22 cells were cultured in normal glucose medium (25 mM d-glucose, I), high-glucose medium (50 mM d-glucose, II), high-glucose medium + 1.25 μm/L SFN (III), high-glucose medium + 2.5 μm/L SFN (IV) or high-glucose medium + 5 μm/L SFN (V) for 0 or 72 h. ROS were measured using the DCFH-DA fluorescent probe under a fluorescence microscope at a magnification of ×400. The fluorescence intensity was correlated with the ROS level. It was observed that the fluorescence intensity was significantly increased in HT22 cells cultured in high glucose medium without SFN (II) compared with the others (I, III, IV, and V) after culturing for 72 h. ROS, reactive oxygen species; DCFH-DA, dichlorodihydrofluorescein-diacetate

High-glucose medium significantly increased DCF fluorescence in HT22 cells at 72 h, and treatment with SFN (from 1.25 to 5.0 µM) induced a significant decrease in DCF fluorescence, which was most obvious after treatment with 2.5 µM SFN. The results above suggest that the neuroprotective effect of SFN are most prominent at a concentration of 2.5 µM (Fig. 7).

## Discussion

**Fig. 8:**
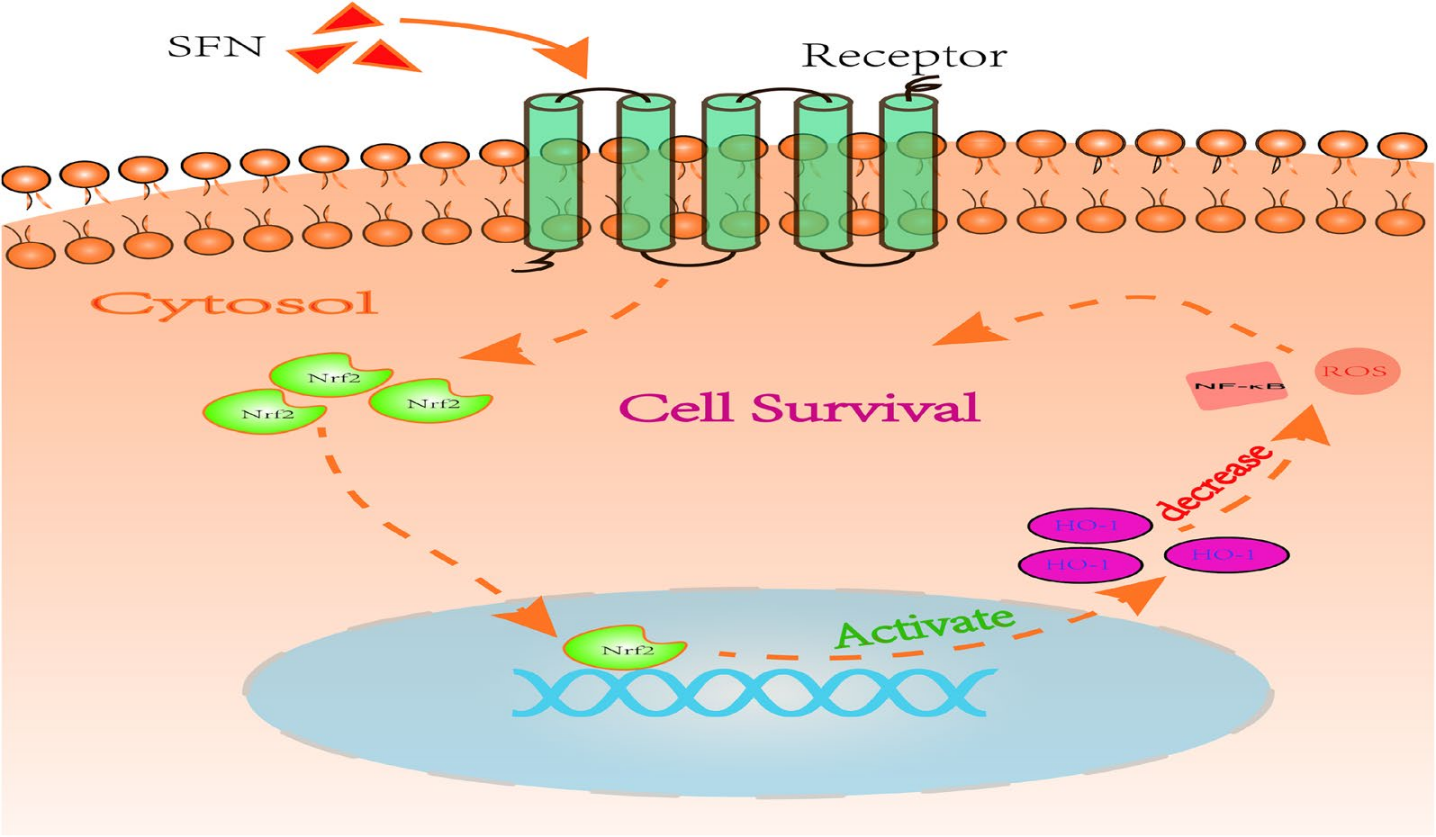
This cartoon depicts the cascade of events in HT22 cells following SFN was added into cell culture medium. SFN binds to its receptor on the cell membrane, causing Nrf2 translocating into nucleus. Nrf2 activated HO-1 expressions by binding to the specific sites on DNA double strand. The activating of Nrf2/HO-1 pathway decreases the productions of NF-κB and ROS, therefore increasing cell survival

We established an in vitro model by exposing HT22 cells to high-glucose medium for 72 h to imitate the chronic hyperglycaemic state of diabetes. We found that high-glucose medium decreased cell viability, increased the generation of ROS, downregulated the expression of Nrf2/HO-1 and upregulated the expression of NF-κB at both the mRNA and protein levels. SFN treatment increased the expression of Nrf2 and downstream effectors in HT22 cells and reversed the increased ROS levels, indicating that the Nrf2 pathway is essential for the maintenance of cell function, which is impaired under hyperglycaemia (Fig. 8).

The brain is especially sensitive to oxidative damage due to its high oxygen consumption rate, its abundant lipid content, and the relative paucity of antioxidant enzymes. Numerous studies have confirmed that diabetes mellitus is associated with enhanced oxidative stress, as characterized by increased lipids, proteins and DNA oxidation products [13]. Mastrocola et al. [14] showed that mitochondria isolated from the brains of STZ-induced diabetic rats present increased ROS levels associated with reduced antioxidant barriers in terms of the GSSG/GSH ratio, GSH peroxidase activity and MnSOD content. This finding indicates that a defect in the Nrf2 signalling pathway may contribute to the increased sensitivity to oxidative stress damage in people with type 2 diabetes. Dermal fibroblasts isolated from genetic diabetic rat models exhibit a state of heightened oxidative stress, while the nuclear accumulation of Nrf2 and the expression of its downstream effectors decrease as the cellular content of Keap1 markedly increases [15]. It has been reported that there is an increase in the generation of ROS/nitrogen species (RNS) in the hippocampus of db/db mice, while less Nrf2 is located in neuronal nuclei [16]. Both the sciatic nerves of STZ-induced diabetic rats and neurons incubated in high-glucose medium exhibit a decrease in the expression of Nrf2 and HO-1 [17]. Although a large number of studies have demonstrated a deficient Nrf2 response in diabetes, the underlying mechanism remains elusive, and further studies to explore the mechanisms of Nrf2 deficiency are required.

Oxidative stress not only can lead to cell damage directly but also may act as a second messenger that activates NF-κB and results in the further expression of proinflammatory cytokines. Under normal conditions, NF-κB is sequestered in the cytoplasm by Iκβ, and oxidative stress caused by hyperglycaemia or other stimuli can activate the Iκβ kinase complex that phosphorylates Iκβ, leading to the activation of NF-κB and inducing the expression of target genes [18]. It has been demonstrated that the hippocampus of STZ-induced diabetic mice presents high levels of ROS and activation of NF-κB, which plays a significant role in neuronal loss and impaired cognitive function [19]. The in vitro results of the present study agree with the in vivo findings that high-glucose medium increases oxidative stress and the inflammatory response in HT22 cells, which may be caused by Nrf2 deficiency.

To verify this hypothesis, we used a pharmacological Nrf2 activator, SFN, which has been shown to exert cellular protection by promoting the nuclear translocation of Nrf2 and thus triggering intracellular defence responses to oxidative stress [20]. Our results showed that high-glucose medium increased the production of ROS and upregulated the expression of NF-κB in HT22 cells. The activation of the Nrf2 pathway using SFN reversed this change. Consistent with our results, early studies also suggested that the pharmacological upregulation of the transcription factor Nrf2 may be a promising strategy to modulate inflammatory reactions in the brain [21]. Nrf2 knockout mice exhibit an exacerbated inflammatory response to LPS, as determined by an increase in microglial density and in the levels of inflammation markers inducible NO synthase, interleukin-6 (IL-6) and tumour necrosis factor-α (TNF-α) compared with those in the hippocampi of wild-type controls [22]. SFN treatment is able to increase the expression of HO-1 and decrease microglial activation and inflammatory cytokine production [23]. Sulforaphane has been reported to decrease interleukin-1 beta (IL-1β) release in Aβ_1-42_-stimulated human microglia-like cells through mechanisms involving the activation of the Nrf2/HO-1 signalling pathway. However, this anti-inflammatory effect of sulforaphane is blunted in cells transfected with a dominant negative Nrf2 construct [21].

There are some limitations to our study. First, the mechanism of Nrf2 deficiency under high-glucose conditions remains unclear. DNA hypermethylation or the activation of glycogen synthase kinase-3β has been reported to exist in hippocampal neurons in AD [24]. Second, we failed to construct RNA interference vectors targeting the Nrf2 or HO-1 genes to test our hypothesis that the activation of Nrf2/HO-1 may suppress inflammation induced by high-glucose conditions. Third, we did not measure the downstream effectors of NF-κB, such as TNF-α, IL-1β and IL-6. Further research is needed to elaborate the association of hyperglycaemia, oxidative stress and inflammation in diabetic encephalopathy.

## Conclusion

Our research indicates that high glucose conditions are associated with increased oxidative stress and inflammation and that the activation of the Nrf2/HO-1 pathway using a pharmacological Nrf2 activator can protect HT22 cells against high-glucose-induced injury.

## Data Availability

The datasets used and/or analysed in the current study are available from the corresponding author on reasonable request.

## Abbreviations

SFN: sulforaphane
Nrf2: NF-E2-related factor 2
HO-1: haem oxygenase-1
NF-κB: nuclear factor-κB
ROS: reactive oxygen species
OD: optical density
DCFH-DA: dichlorodihydrofluorescein-diacetate
